# Successful Live Birth Following PGT‐SR in a Couple Who Were Both Carriers of Balanced Reciprocal Translocations Identified During Recurrent Pregnancy Loss Workup: A Case Report

**DOI:** 10.1002/rmb2.70022

**Published:** 2026-01-29

**Authors:** Hiroaki Negishi, Daisuke Higeta, Satoh Maki, Setsuko Satoh, Youko Matsumiya, Nanami Takano, Hidemi Yokota, Mikako Yokota, Yoshimasa Yokota

**Affiliations:** ^1^ Reproductive Medicine Yokota Maternity Hospital Maebashi Gunma Japan; ^2^ Perinatal Medical Center Gunma University Hospital Maebashi Gunma Japan

**Keywords:** balanced reciprocal translocation, chromosomal segregation, embryo biopsy, PGT‐SR, preimplantation genetic testing, recurrent pregnancy loss

## Abstract

**Case:**

A non‐consanguineous couple with recurrent pregnancy loss was found to be carriers of two different balanced reciprocal translocations: 46,XX,t(13;16)(q14;q23) and 46,XY,t(4;12)(q21.1;q15). They underwent IVF with PGT‐SR, from which seven blastocysts were obtained.

**Outcome:**

Only one embryo was identified as both euploid and structurally balanced. This embryo was transferred in a hormone replacement cycle, resulting in a successful pregnancy and the birth of a healthy male infant who inherited both parental balanced translocations.

**Conclusion:**

This case demonstrates the feasibility and utility of PGT‐SR in rare dual‐carrier cases, underscoring the importance of tailored genetic counseling, careful embryo selection, and realistic prognostic discussion for couples with complex chromosomal abnormalities.

## 1 Introduction

Chromosomal abnormalities are detected in approximately 4%–5% of couples experiencing recurrent pregnancy loss (RPL), with most cases involving balanced structural rearrangements [1, 2, 3, 4]. Reciprocal translocation carriers (RTL) are estimated to occur in 1 in 400 individuals in the general population, making the incidence of couples where both partners are RTL carriers exceedingly rare. Gametes with unbalanced chromosomal content resulting from such translocations can lead to infertility, recurrent miscarriages, or offspring with congenital anomalies [5, 6, 7]. Despite its low frequency, the occurrence of live births with unbalanced chromosomal aberrations accompanied by congenital anomalies has been documented in natural pregnancies involving RTL carriers (0.4%–2.9%). Therefore, caution should be exercised even after conception is achieved [2, 6, 8, 9, 10]. When a chromosomal abnormality such as a balanced translocation is identified in either partner, genetic counseling is essential to explain the probability of achieving a chromosomally balanced or normal fetus, as well as the pros and cons of preimplantation genetic testing for structural rearrangements (PGT‐SR). While RTL carriers may achieve live births in 60%–80% of cases [11, 12, 13, 14], current evidence does not support a clear benefit of PGT‐SR in increasing live birth rates and may even suggest lower success in some reports [15, 16]. Nevertheless, PGT‐SR offers the potential advantage of detecting embryos with unbalanced chromosomal rearrangements.

We report a rare case in which both partners were diagnosed as balanced reciprocal translocation carriers during RPL workup. Among seven blastocysts obtained through ART, only one was identified as transferable via PGT‐SR, resulting in a successful pregnancy and live birth. However, the newborn's peripheral lymphocyte karyotype showed that both balanced translocations had been transmitted to him.

## 2 Case Report

**FIGURE 1:**
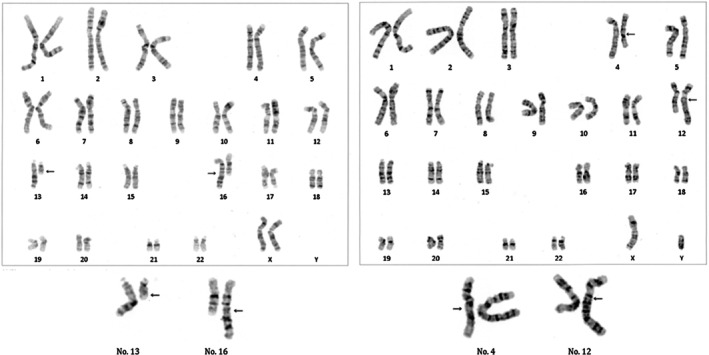
Parental karyotyping results. Karyotypes of both partners obtained from peripheral blood lymphocyte analysis. The female partner exhibited a balanced reciprocal translocation: 46,XX,t(13;16)(q14;q23), and the male partner carried a different balanced reciprocal translocation: 46,XY,t(4;12)(q21.1;q15).

**FIGURE 2:**
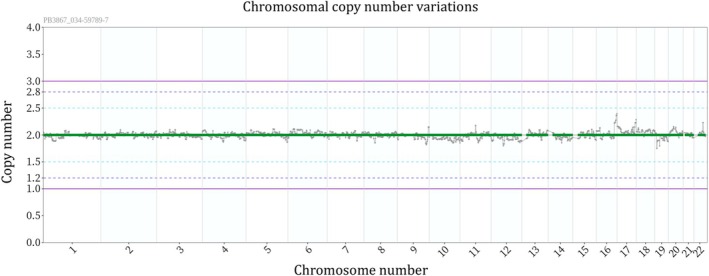
Results of PGT‐SR in blastocyst (ID 7). Results of PGT‐SR for embryo (ID 7) showing chromosomal copy number variations. Under the ethical regulations of the Japan Society of Obstetrics and Gynecology, the embryo's sex is withheld from both physicians and patients.

**FIGURE 3:**
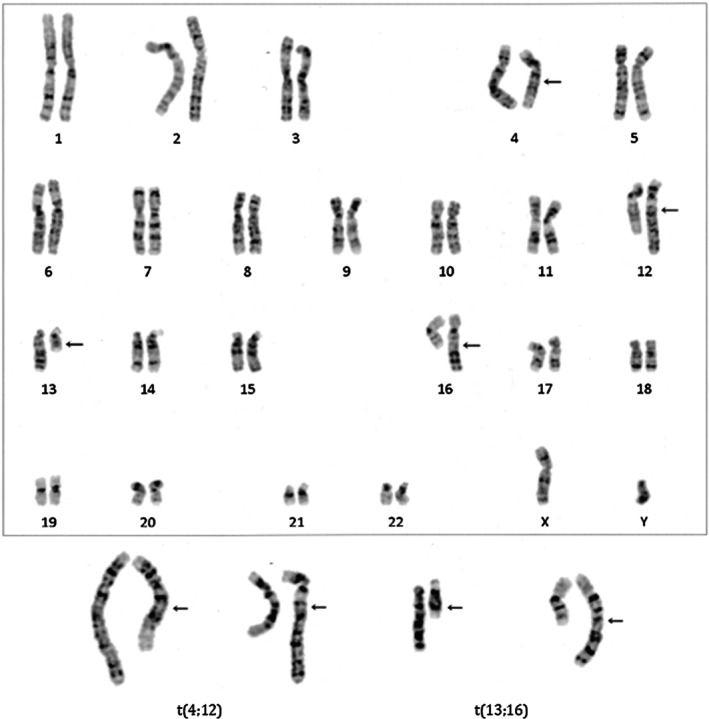
Postnatal karyotyping result. Postnatal G‐band karyotyping of peripheral lymphocytes is 46,XY, t(4;12)(q21.1;q15)pat, t(13;16)(q14;q23)mat. The baby inherited all parental reciprocal translocations.

A 34‐year‐old woman (menarche at age 15, gravida 2, para 0) and her 34‐year‐old husband were referred to our clinic after two miscarriages at 8 and 9 weeks gestation, respectively. Both partners had unremarkable medical and family histories. Her BMI was 19.1 kg/m^2^, and she had irregular menstrual cycles. Transvaginal ultrasound revealed no abnormalities in the uterus or adnexa. Hormonal profile in serum during menstruation revealed: LH: 7.7 mIU/mL, FSH: 7.7 mIU/mL, estradiol: 34 pg/mL, PRL: 10.6 ng/mL, testosterone: 18 ng/dL, AMH: 6.94 ng/mL. Recurrent pregnancy loss evaluation in serum showed: TSH: 1.0 μIU/mL, free‐thyroxine: 1.04 ng/dL, HbA1c: 5.1%, protein S activity: 60%, protein S antigen: 63%, factor XII: 65%, negative for antiphospholipid antibodies and infections (e.g., Chlamydia IgG). Karyotyping of peripheral lymphocytes revealed (Figure 1): female: 46,XX,t(13;16)(q14;q23), male: 46,XY,t(4;12)(q21.1;q15). High‐resolution breakpoint mapping (e.g., FISH confirmation or sequencing‐based junction analysis) was not performed; therefore, simplified schematic illustrations are provided in the Supporting Information to aid visualization of the translocation structure and quadrivalent formation (Figure S1A,B). After genetic counseling, the couple elected to proceed with ART and PGT‐SR. Thirty oocytes (26 mature oocytes) were retrieved using a progestin‐primed ovarian stimulation protocol. Fourteen oocytes underwent conventional IVF (12 fertilized) and 12 underwent ICSI (7 fertilized). Seven blastocysts were biopsied and analyzed by PGT‐SR. Biopsied trophectoderms were subjected to whole genome amplification and library preparation using the EmbryoMap Kit (Vitrolife, Sweden), following the manufacturer's protocol. Sequencing was performed using 36 bp paired‐end reads on an Illumina MiSeq platform. Chromosomal copy number variations (CNVs) and structural rearrangements were analyzed using the eMap software (Vitrolife, Sweden). Embryos with mosaicism of 20% or greater were classified as mosaic, while those with abnormal copy number in 80% or more of cells were classified as aneuploid. Ploidy status was assessed by genotyping single nucleotide variants (SNVs) and evaluating allele ratios. All genetic testing was conducted by Varinos Inc. (Tokyo, Japan). Only one embryo (ID 7, ICSI‐derived 3BA) was diagnosed as exhibiting chromosomally balanced or euploid status for all autosomes (Figure 2). The other six embryos were diagnosed as harboring autosomal aneuploidy and/or structural abnormalities (Table 1). Among them, four exhibited unrelated aneuploidies, including monosomy 8, trisomy 13, monosomy 19, and partial trisomy 17; one embryo had monosomy 19 as the sole abnormality. Unbalanced translocations involving one or both parental rearrangements were observed in five embryos (Table 2). Segregation patterns were classified as alternate, adjacent I, adjacent II, or 3:1 types (Table 3). ID 7 embryo was transferred during a hormone replacement cycle. Pregnancy was confirmed and progressed uneventfully. The couple declined amniocentesis after counseling. At 40 weeks and 1 day, a healthy male infant was delivered via cesarean section (birth weight 3033 g, Apgar scores 8/9/9). Postnatal G‐band karyotyping of peripheral lymphocytes is 46,XY, t(4;12)(q21.1;q15)pat, t(13;16)(q14;q23)mat (Figure 3).

**TABLE 1:** Summary of chromosomal findings from seven blastocysts subjected to PGT‐SR.

Embryo ID	Maternal translocation;46,XX,t(13;16)(q14;q23)					Paternal translocation;46,XY,t(4;12)(q21.1;q15)					Aneuploidy findings		
	Segregation type	Chromosome number	Partial duplication	Chromosome number	Partial deletion	Segregation type	Chromosome number	Partial duplication	Chromosome number	Partial deletion	Aneuploidy	High‐level mosaicism	Blastocyst grade
1	Alternate		—		—	Adjacent II	12	pter‐q21.1	4	pter‐q21.22	T 8		3AB
2	3:1 Segregation	13	q14.1;q23		—	3:1 Segregation		—	4	pter‐q21.22	Te 13		3AA
									12	q21.1‐qter			
3	3:1 Segregation	13	q14.1;q23		—	Adjacent I	12	q21.1‐qter	4	q21.23‐qter		M 18	4AB
4	Adjacent II	16	pter‐q22.1	13	pter‐q13.1	Adjacent I	4	q21.23‐qter	12	q21.1‐qter			3BA
5	Alternate		—		—	Alternate		—		—	M 19		3AA
6	3:1 Segregation		—	13	pter‐q13.1	Adjacent II	12	pter‐q21.1	4	pter‐q21.22	Partial T 17	T 1, T 14	3BB
				16	q22.3‐qter						p13.3‐q11.2	Partial T12 partial T 13	
7	Alternate		—		—	Alternate		—		—			3BA

Abbreviations: M; monosomy; T; trisomy, Te; tetrasomy.

**TABLE 2:** Chromosomal origin of unbalanced embryos and blastocyst grading.

Translocation chromosome		Other chromosome	Embryo number	Blastocyst grade
Maternal 13;16	Paternal 4;12			
Balanced	Balanced	Euploid	1	3BA^c^
		Aneuploid	1	3AA
Balanced	Unbalanced	Aneuploid	1	3AB
Unbalanced	Unbalanced	Euploid	1	3BA
		Aneuploid^a^	2	3AA, 3BB
		Aneuploid (mosaic)^b^	1	4AB

^a^ Include partial trisomy.

^b^ High level mosaicism.

^c^ Transferred embryo.

**TABLE 3:** Segregation types observed in embryos according to translocation origin.

Segregation type	Translocation chromosome	
	Maternal 13;16	Paternal 4;12
Alternate	3	2
Adjacent I	0	2
Adjacent II	1	2
3:1 Segregation	3	1

## 3 Discussion

Chromosomal abnormalities are detected in approximately 4%–5% of couples with recurrent pregnancy loss (RPL), with reciprocal translocations (RTL) accounting for 60%–70%, Robertsonian translocations for 10%–20%, and inversions (excluding chromosome 9 pericentric inversion) for approximately 10% [1, 2, 3, 4]. Although the live birth rate in natural pregnancies among RTL carriers is lower than that of couples with normal karyotypes (70%–80%), it is still reported to be around 50%–60% in RTL carriers [3, 4]. Some reports even suggest that the healthy live birth rate in all structural chromosomal abnormality cases reaches 83%, which is not significantly different from that of controls [6]. Previous reviews have shown that PGT‐SR does not provide clear benefits over natural conception [15], suggesting its limited utility for RPL cases [15, 16, 17, 18, 19, 20]. Therefore, comprehensive genetic counseling is essential to communicate both the potential benefits and limitations of PGT‐SR to patients. The general prevalence of RTL is reported to be 0.16%–0.2% [4], making the probability of both partners being RTL carriers extremely rare (0.026%–0.04%). To our knowledge, only one report by Liu et al. [21] has documented a non‐consanguineous couple who were both RTL carriers and underwent PGT‐SR leading to pregnancy and live birth. To substantiate the rarity of couples in which both partners carry balanced reciprocal translocations, we conducted a literature search in PubMed (accessed on 9 January 2026) using combinations of the following terms: “reciprocal translocation,” “both partners,” “both carriers,” “dual carrier/double carrier,” and “PGT‐SR/PGD.” This search did not identify any additional reports describing a non‐consanguineous couple in which both partners were reciprocal translocation carriers undergoing PGT‐SR leading to live birth, beyond the case reported by Liu et al. Therefore, the available indexed literature supports that dual‐carrier reciprocal translocation couples undergoing PGT‐SR are exceptionally rare. In our case, 26 mature oocytes were obtained via ART, with 19 successfully fertilized (fertilization rate 73%). Seven embryos reached the blastocyst stage (blastocyst formation rate per oocyte: 27%, per fertilized embryo: 37%). These rates are comparable to findings by Xie et al. [17] (32.9% blastocyst formation) and Wang et al. (37.9%) [18], though lower than the ~60% reported for patients under 39 years old in the general ART population [19]. This may be due to early developmental arrest of unbalanced embryos. Ziravard et al. [20] reported a higher rate of alternate segregation at Day 5/6 than at Day 3 (45% vs. 34%) and a significant decrease in 3:1 segregation (4% vs. 28%, *p* < 0.0001), suggesting embryo‐level selection occurs as development progresses. Of the seven blastocysts analyzed in our case, only one was euploid and structurally balanced. Aneuploidy unrelated to the translocations was observed in five embryos (71%) (Table 2), a relatively high rate given the maternal age of 34. Only one embryo exhibited isolated aneuploidy (monosomy 19), while three others had combined unbalanced translocations, making interchromosomal effects unlikely. Four embryos showed unbalanced structural abnormalities involving both translocations, and one involved a single translocation. Segregation types included adjacent II and 3:1 on the maternal side, and adjacent I/II and 3:1 on the paternal side (Table 3). 3:1 segregation was more common in oocyte‐derived embryos, while adjacent segregation dominated in sperm‐derived embryos (not significant, *p* = 0.206). According to Daniel's triangle by Cohen et al. [22], none of these embryos were predicted to result in viable offspring (Table 1). Factors influencing meiotic segregation of quadrivalents include parental sex and age, breakpoint location (terminal breakpoint [20], translocated segment ratio [17], involved arms [18]), chromosome type (acrocentric), and quadrivalent asymmetry. Xie et al. [17] found that smaller translocated segments relative to the chromosome arm reduced the odds of unbalanced embryos (OR 0.81, *p* = 0.004), while female carriers and acrocentric involvement increased the odds (OR 1.29 and 1.21, *p* < 0.001, respectively). In the present case, breakpoints were defined by G‐banding without high‐resolution breakpoint mapping; thus, we provide simplified schematic illustrations (Figure S1A,B) to facilitate visualization of the derivative chromosome configuration and quadrivalent formation. Wang et al. reported that translocated segment ratios < 0.2 favored alternate segregation, while ratios ≥ 0.2 increased adjacent II segregation (6.7% vs. 15.5%, *p* = 0.013). Acrocentric chromosomes also showed a higher frequency of 3:1 segregation (24.8% vs. 5.1%, *p* < 0.0001), with no significant sex‐related differences [18]. Ziravard et al. demonstrated that Day 3 embryos showed significantly higher 3:1 segregation, which decreased by the blastocyst stage. Terminal breakpoints may interfere with proper crossover and increase unbalanced outcomes. Their study showed that alternate and adjacent I segregation increased from Day 3 to Day 5/6, suggesting selection against more severely unbalanced embryos [20]. Our case supports this, as three of seven embryos (43%) showed 3:1 segregation and involved maternal terminal breakpoints. Liu et al. [21] reported a 30‐year‐old woman with secondary infertility and three consecutive miscarriages, later diagnosed as an RTL carrier couple. Out of 16 retrieved oocytes, 10 fertilized, and three blastocysts were biopsied. Two were normal/balanced, one was unbalanced, and no additional aneuploidies were found. Among RTL carriers, approximately 60%–70% of blastocysts are expected to be unbalanced [1, 2, 3], leaving 30%–40% as normal/balanced [3, 23, 24]. Therefore, when both partners are carriers, the probability of obtaining a normal/balanced embryo is 9%–16%. However, since some embryos also carry unrelated aneuploidies, the actual rate of transferable embryos is even lower. According to 2023 Japanese data of PGT‐SR, only 18.4% were euploid and 25.6% euploid and mosaicism. This implies that in dual‐RTL cases, the chance of obtaining an embryo that is both structurally balanced and euploid (including mosaicism) is approximately 6.6% (= 25.6% × 25.6%). To further strengthen this theoretical estimate, we provide a quantitative framework combining the probability of alternate segregation for each translocation (*p*ₐ for the maternal translocation and *p*ᵦ for the paternal translocation) with the maternal age–dependent euploid rate (*q*) for chromosomes unrelated to the translocations. Under the simplifying assumption that these events are approximately independent, the expected probability of obtaining an embryo that is both structurally balanced and euploid can be expressed as *P* = *p*ₐ × *p*ᵦ × *q*. This framework clarifies why the proportion of transferable embryos can be markedly reduced in dual‐carrier couples and underscores the need for an adequate number of oocytes/embryos to obtain at least one transferable blastocyst. In our case, one of seven embryos (14%) was transferable, aligning with the predicted 9%–16% range. Although the benefit of PGT‐SR over natural conception remains unclear, a sufficient number of retrieved oocytes is essential to increase the likelihood of obtaining a transferable embryo. Back‐calculating from our data, 6–15 blastocysts, 15–38 fertilized embryos, and 21–54 oocytes would be required to yield one transferable embryo, assuming a 6.6%–16.6% transferability rate. Differences in reported outcomes may be influenced by breakpoint characteristics, carrier age, reason for karyotyping, and chromosome types. Our analysis includes only successful cases with transferable embryos, and further studies should also include failed cycles. Clinicians must provide realistic prognostic information to RTL carrier couples. Establishing a nationwide registry system for PGT‐SR outcomes would help accumulate data and improve patient counseling. In Japan, since the introduction of PGD for recurrent pregnancy loss in 2006, PGT‐SR and related procedures have been performed for more than a decade under case‐by‐case ethical review at individual institutions. These earlier datasets may contain detailed structural information—such as translocated segment ratios, breakpoint characteristics, and predicted segregation modes—which could serve as a valuable resource for reanalyzing correlations between translocation architecture and clinical outcomes. Future efforts to harmonize and integrate these historical data with a nationwide registry would further strengthen evidence generation and prognostic counseling.

In our case, the male baby inherited all parental reciprocal translocations. Current CNV analysis in PGT‐SR cannot distinguish carrier from normal embryos. Shi et al. showed that SNP‐based haplotype analysis, such as Mapping Allele with Resolved Carrier Status (MaReCs) technology, can differentiate carrier from noncarrier embryos, highlighting the need for future development [25]. Rossiet al. reported that a sperm selection process using the hypoosmotic swelling test (HOST) allows the proportion of unbalanced spermatozoa to drop to 15% in a complex reciprocal translocation carrier [26]. When the carrier is male, this method could potentially provide an effective means of sperm selection in cases of reciprocal translocation.

We report a rare case of a couple in which both partners were carriers of balanced reciprocal translocations. Of seven blastocysts analyzed by PGT‐SR, only one was transferable and led to the birth of a healthy child following embryo transfer in a hormone replacement cycle. For effective counseling, physicians should be equipped with accurate prognostic data considering age, sex, and translocation characteristics. To this end, accumulation of case reports and implementation of a national data collection system are strongly encouraged.

## Ethics Statement

This case report was approved by the Ethics Committee of Yokota Maternity Hospital (approval number YKM‐2024‐09). All procedures performed in studies involving human participants were in accordance with the ethical standards of the institutional and/or national research committee and with the 1964 Helsinki declaration and its later amendments or comparable ethical standards.

## Consent

Informed consent was obtained from all individual participants included in the study.

## Conflicts of Interest

The authors declare no conflicts of interest.

## Supporting information


**Figure S1:** (A) Simplified schematic representation of parental reciprocal translocations. Breakpoints (★) are indicated schematically and are not drawn to scale. (B) Schematic illustration of quadrivalent formation during meiosis. Homologous chromosome segments derived from chromosomes 13 and 16 (female) and chromosomes 4 and 12 (male) align to form a quadrivalent configuration during meiosis. Breakpoints (★) are indicated schematically and are not drawn to scale.

## Data Availability

The data that support the findings of this study are available from the corresponding author upon reasonable request. The data are not publicly available due to privacy or ethical restrictions.

## References

[1] C. K. Lim , J. W. Cho , I. O. Song , I. S. Kang , Y. D. Yoon , and J. H. Jun , “Estimation of Chromosomal Imbalances in Preimplantation Embryos From Preimplantation Genetic Diagnosis Cycles of Reciprocal Translocations With or Without Acrocentric Chromosomes,” Fertility and Sterility 90, no. 6 (2008): 2144–2151, 10.1016/j.fertnstert.2007.10.035. 18440525

[2] C. S. Ottolini , L. J. Newnham , A. Capalbo , et al., “Genome‐Wide Maps of Recombination and Chromosome Segregation in Human Oocytes and Embryos Show Selection for Maternal Recombination Rates,” Nature Genetics 47, no. 7 (2015): 727–735, 10.1038/ng.3306. 25985139 PMC4770575

[3] S. Zhang , C. Lei , J. Wu , et al., “Analysis of Segregation Patterns of Quadrivalent Structures and the Effect on Genome Stability During Meiosis in Reciprocal Translocation Carriers,” Human Reproduction 33, no. 4 (2018): 757–767, 10.1093/humrep/dey036. 29579270

[4] S. Alfarawati , E. Fragouli , P. Colls , and D. Wells , “First Births After Preimplantation Genetic Diagnosis of Structural Chromosome Abnormalities Using Comparative Genomic Hybridization and Microarray Analysis,” Human Reproduction 26, no. 6 (2011): 1560–1574, 10.1093/humrep/der068. 21447693

[5] P. N. Scriven , A. H. Handyside , and C. M. Ogilvie , “Chromosome Translocations: Segregation Modes and Strategies for Preimplantation Genetic Diagnosis,” Prenatal Diagnosis 18, no. 13 (1998): 1437–1449. 9949444

[6] S. J. Morin , J. Eccles , A. Iturriaga , and R. S. Zimmerman , “Translocations, Inversions and Other Chromosome Rearrangements,” Fertility and Sterility 107, no. 1 (2017): 19–26, 10.1016/j.fertnstert.2016.10.013. 27793378

[7] N. Fatemi , M. Varkiani , F. Ramezanali , et al., “Risk Factors Associated With Recurrent Pregnancy Loss and Outcome of Pre‐Implantation Genetic Screening of Affected Couples,” International Journal of Fertility & Sterility 15, no. 4 (2021): 269–274, 10.22074/IJFS.2021.137626.1027. 34913295 PMC8530214

[8] M. T. Franssen , J. C. Korevaar , F. van der Veen , N. J. Leschot , P. M. Bossuyt , and M. Goddijn , “Reproductive Outcome After Chromosome Analysis in Couples With Two or More Miscarriages: Case‐Control Study,” BMJ (Clinical Research Ed.) 332 (2006): 759–763. 10.1136/bmj.38735.459144.2F PMC1420685 16495333

[9] M. Sugiura‐Ogasawara , Y. Ozaki , T. Kitaori , K. Kumagai , and S. Suzuki , “Midline Uterine Defect Size Is Correlated With Miscarriage of Euploid Embryos in Recurrent Cases,” Fertility and Sterility 93 (2010): 1983–1988. 19249757 10.1016/j.fertnstert.2008.12.097

[10] J. C. Barber , A. E. Cockwell , E. Grant , S. Williams , R. Dunn , and C. M. Ogilvie , “Is Karyotyping Couples Experiencing Recurrent Miscarriage Worth the Cost?,” BJOG: An International Journal of Obstetrics and Gynaecology 117 (2010): 885–888. 20482539 10.1111/j.1471-0528.2010.02566.x

[11] M. D. Stephenson and S. Sierra , “Reproductive Outcomes in Recurrent Pregnancy Loss Associated With a Parental Carrier of a Structural Chromosome Rearrangement,” Human Reproduction 21 (2006): 1076–1082. 16396938 10.1093/humrep/dei417

[12] M. Sugiura‐Ogasawara , Y. Ozaki , T. Sato , N. Suzumori , and K. Suzumori , “Poor Prognosis of Recurrent Aborters With Either Maternal or Paternal Reciprocal Translocations,” Fertility and Sterility 81 (2004): 367–373. 14967375 10.1016/j.fertnstert.2003.07.014

[13] M. Goddijn , J. H. Joosten , A. C. Knegt , et al., “Clinical Relevance of Diagnosing Structural Chromosome Abnormalities in Couples With Repeated Miscarriage,” Human Reproduction (Oxford, England) 19 (2004): 1013–1017. 14990541 10.1093/humrep/deh172

[14] H. Carp , B. Feldman , G. Oelsner , and E. Schiff , “Parental Karyotype and Subsequent Live Births in Recurrent Miscarriage,” Fertility and Sterility 81 (2004): 1296–1301. 15136093 10.1016/j.fertnstert.2003.09.059

[15] M. Sugiura , “Current Status of Preimplantation Genetic Test and Prenatal Test,” Journal of the Japan Medical Association 137 (2008): 49–52.

[16] S. Ikuma , T. Sato , M. Sugiura‐Ogasawara , M. Nagayoshi , A. Tanaka , and S. Takeda , “Preimplantation Genetic Diagnosis and Natural Conception: A Comparison of Live Birth Rates in Patients With Recurrent Pregnancy Loss Associated With Translocation,” PLoS One 10, no. 6 (2015): e0129958, 10.1371/journal.pone.0129958. 26083495 PMC4470686

[17] P. Xie , L. Hu , Y. Peng , et al., “Risk Factors Affecting Alternate Segregation in Blastocysts From Preimplantation Genetic Testing Cycles of Autosomal Reciprocal Translocations,” Frontiers in Genetics 13 (2022): 880208, 10.3389/fgene.2022.880208. 35719400 PMC9201810

[18] J. Wang , D. Li , Z. Xu , et al., “Analysis of Meiotic Segregation Modes in Biopsied Blastocysts From Preimplantation Genetic Testing Cycles of Reciprocal Translocations,” Molecular Cytogenetics 12 (2019): 11, 10.1186/s13039-019-0423-7. 30858883 PMC6390622

[19] H. Kikuchi , Y. Nagase , Y. Goto , S. Okitsu , K. Taniguchi , and T. Kobayashi , “Multi‐Center Investigation of the Culture Status, Survival Rate After Thawing and Pregnancy Rate for Human Conventional‐IVF and ICSI—2023,” Journal of Clinical Embryologists 26 (2024): 1–10.

[20] N. T. Ziravard , I. L. Puppo , A. F. Saifitdinova , T. V. Vavilova , and A. S. Glotov , “Assessment of Quadrivalent Characteristics Influencing Chromosome Segregation by Analyzing Human Preimplantation Embryos From Reciprocal Translocation Carriers,” Comparative Cytogenetics 18 (2024): 1–13, 10.3897/compcytogen.18.115070. 38298496 PMC10825968

[21] D. Liu , C. Chen , X. Zhang , et al., “Successful Birth After Preimplantation Genetic Testing for a Couple With Two Different Reciprocal Translocations and Review of the Literature,” Reproductive Biology and Endocrinology 19 (2021): 58, 10.1186/s12958-021-00731-2. 33879178 PMC8056626

[22] O. Cohen , C. Cans , M. A. Mermet , J. Demongeot , and P. Jalbert , “Viability Thresholds for Partial Trisomies and Monosomies: A Study of 1,159 Viable Unbalanced Reciprocal Translocations,” Human Genetics 93, no. 2 (1994): 188–194, 10.1007/BF00210608. 8112744

[23] M. Ding , Y. T. Zhang , Y. Sun , F. Lin , Z. Diao , and J. Zhou , “A Mathematical Model for Predicting the Number of Transferable Blastocysts in Next‐Generation Sequencing‐Based Preimplantation Genetic Testing,” Archives of Gynecology and Obstetrics 305 (2022): 241–249, 10.1007/s00404-021-06050-6. 34218301

[24] L. Walters‐Sen , D. Neitzel , R. E. Ellsworth , S. Poll , N. Faulkner , and S. Aradhya , “Derivative and Non‐Derivative Aneuploidy Rates in PGT‐Tested Blastocysts From Carriers of Structural Rearrangements,” Reproductive Biomedicine Online 50, no. 3 (2025): 104407. 39939197 10.1016/j.rbmo.2024.104407

[25] H. Shi , W. Niu , H. Bai , et al., “Preimplantation Genetic Testing and Carrier Status Detection in Patients With Balanced Chromosomal Rearrangements: A Real‐World Multicenter Retrospective Study,” Fertility and Sterility 124 (2025): 297–306. 40246052 10.1016/j.fertnstert.2025.04.008

[26] C. Rossi , J. P. Siffroi , L. Ruosso , et al., “Chromosomal Segregation Analysis and HOSTbased Sperm Selection in a Complex Reciprocal Translocation Carrier,” Journal of Assisted Reproduction and Genetics 40 (2023): 33–40. 36441422 10.1007/s10815-022-02665-z PMC9840725

