# Supplementary material for: Successful Live Birth Following PGT‐SR in a Couple Who Were Both Carriers of Balanced Reciprocal Translocations Identified During Recurrent Pregnancy Loss Workup: A Case Report
Source: Reprod Med Biol. 2026 Jan 29;25(1):e70022. doi: 10.1002/rmb2.70022 (PMC12853316; doi:10.1002/rmb2.70022)
Supplement: Supplementary file 1 — Figure S1: (A) Simplified schematic representation of parental reciprocal translocations. Breakpoints (★) are indicated schematically and are not drawn to scale. (B) Schematic illustration of quadrivalent formation during meiosis. Homologous chromosome segments derived from chromosomes 13 and 16 (female) and chromosomes 4 and 12 (male) align to form a quadrivalent configuration during meiosis. Breakpoints (★) are indicated schematically and are not drawn to scale. [file RMB2-25-e70022-s001.zip › rmb270022-sup-0001-FigureS1A.docx]

Figure S1A. Simplified schematic representation of parental reciprocal translocations

Breakpoints (★) are indicated schematically and are not drawn to scale.

ch4

ch12

Male:46,XY,t(4;12)(q21.1;q15)

**〇**

**〇**

**〇**

**〇**


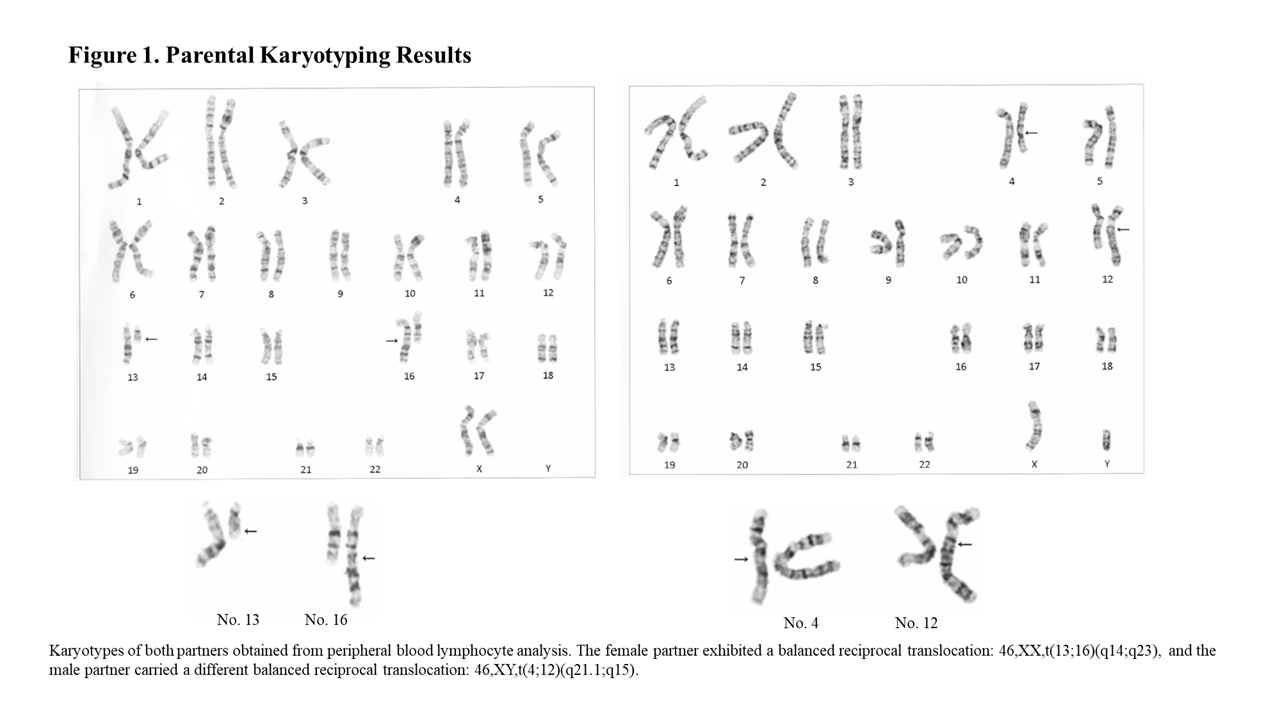


★

★

der(4)

der(12)

★ breakpoint

ch13

ch16

Female: 46,XX,t(13;16)(q14;q23)

**〇**

**〇**


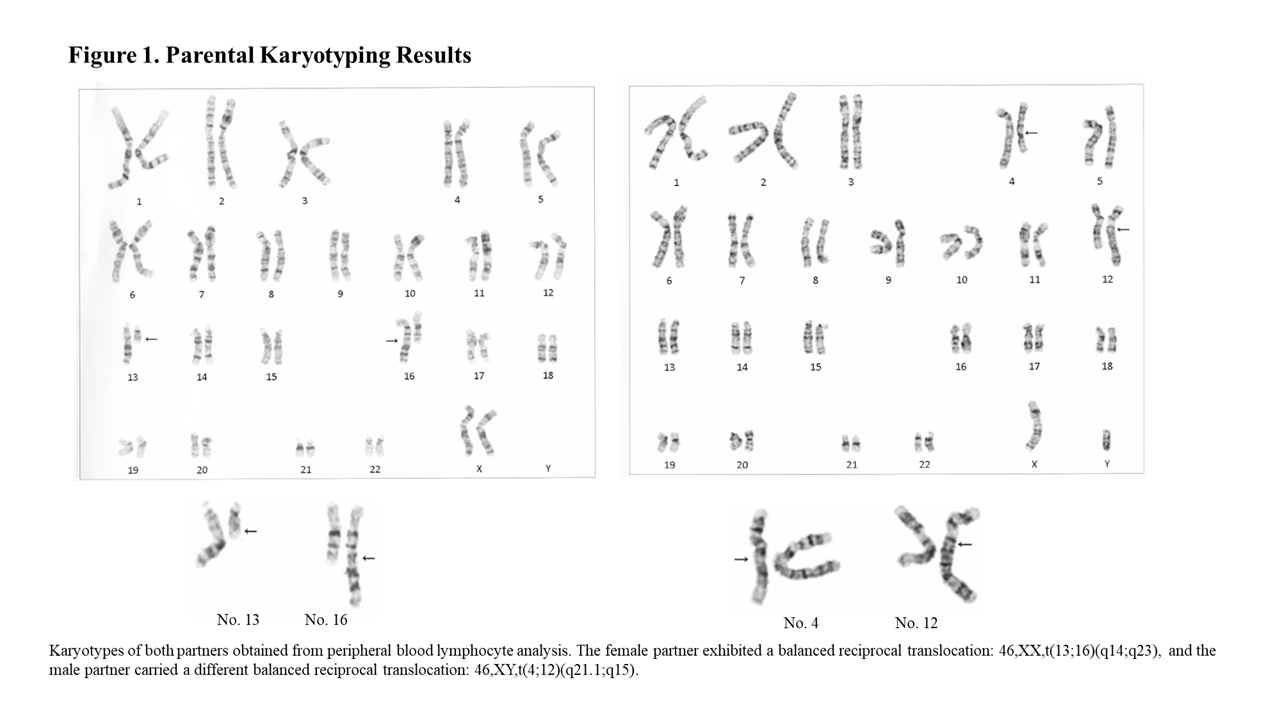


★

der(13)

**〇**

**〇**

★

der(16)
